# An extraordinary palaeontinid from the Triassic of Korea and its significance

**DOI:** 10.1038/srep40691

**Published:** 2017-01-18

**Authors:** Kye Soo Nam, Ying Wang, Dong Ren, Jong Heon Kim, Jacek Szwedo

**Affiliations:** 1 Daejeon Science High School for the Gifted, Daejeon 34142, Republic of Korea; 2 Beijing Museum of Natural History, 126 Tianqiao South St, Beijing, 100050, PR China; 3 Key Lab of Insect Evolution and Environmental Changes, Capital Normal University, Beijing, 100048, PR China; 4 Department of Earth Science Education, Kongju National University, 56 Gongjudaehak-ro Gongju-si 32588, Republic of Korea; 5 Department of Invertebrate Zoology and Parasitology, University of Gdańsk, 59, Wita Stwosza Street, PL80-308 Gdańsk, Poland

## Abstract

A new, extraordinary palaeontinid *Hallakkungis amisanus* Nam, Wang & Szwedo, gen. et sp. nov., from the Upper Triassic of the Amisan Formation in Boryeong City, Korea is described. It is the first Palaeontinidae from Korea. The newly described taxon displays a mosaic of characters present in presumed ancestors of this insect family and some highly advanced features.

The first record of the Late Triassic palaeontinid from Korea presented below comes from the upper deposits of the Nampo Group. Palaeontinidae is an extinct family of cicadomorphan hoppers (Hemiptera: Cicadomorpha), superficially resembling huge moths, which existed since the Triassic to end of the Late Cretaceous in Europe, Asia, and South America. Palaeontinids had large bodies covered with bristles (setae), small heads and broad wings. The host plants of these plant-sucking insects have been assumed to be ginkgophytes based on the geographic distribution of both groups.

The Nampo Group is a non-marine deposit and mainly distributed in Boryeong City and Cheonyang-gun County, the western part of Chungcheongnam-do Province in Korea. The Nampo Group is distributed in the Chungnam Basin of southwestern part of the Chungcheongnam-do, and consists of a 3,000 m-thick sequence of terrestrial sediments^1^,^2^,^3^. The Chungnam Basin contains several subbasins^4^,^5^; the insectiferous locality is located in the Oseosan Subbasin (Fig. 1a–d). The fossil comes from the Upper Triassic, Norian (*ca*. 227 – *ca*. 208.5 Mya) Amisan Formation, which is one of five formations in the Nampo Group^6^.

Abundant fossil plants have been found from the Amisan and the Baegunsa formations, and fossil wood from the Jogyeri Formation^7^,^8^,^9^. The Nampo leaf floras based on material from the Amisan and the Baegunsa formations were described, found similar to each other^10^ and assigned to the *Dictyophyllum-Clathropteris* type of palaeoflora^10^. This flora is known from the southern part of China from the Late Triassic to Early Jurassic^11^, with more occasional occurrences as far north as the Korean Peninsula, and characterized by plants typical of the tropical to subtropical climate^10^,^12^.

The specimen under study was collected from the Chungnam Basin of the Boryeong City, South Korea. The Boryeong deposits consist of dark shale, sandstone, siltstone, and conglomerate. The age of this insect fauna is still debatable, but it is generally considered to be Late Triassic or Early Jurassic^4^,^5^. We consider it as Late Triassic in age based on the analysis of the fossil conchostracans (*Estherites kawasaki*) and plants (*Equisetites ferganensis*).

It is the first record of Palaeontinidae from Korea, for the moment the oldest record of the family and one of the first reports of fossil insects from this locality – only *Mesopsyche dobrokhotovae* Novokshonov, 1997 (Mecoptera: Mesopsychidae) was listed recently^13^. There are a few other insect remains representing Hemiptera, Coleoptera, Ephemeroptera, Blattodea, Plecoptera, Grylloblattodea and Mecoptera found there.

## Material and Method

This specimen was examined with a dissecting microscope (Nikon SMZ 800) and illustrated with the aid of a drawing tube attached to the microscope. Line drawing of tegmen was compiled using Adobe Photoshop CS graphic software. Fossil photograph was taken using a digital camera (Nikon D700).

The type material is deposited in the Department of Earth Science Education, Kongju National University, Korea. The wing venation nomenclature of Palaeontinidae used in this paper is based on the interpretations by Wang B. *et al*.^14^ and Nel *et al*.^15^.

**Systematic Palaeontology**

Order HEMIPTERA Linnæus, 1758

Suborder CICADOMORPHA Evans, 1946

Superfamily PALAEONTINOIDEA Handlirsch, 1906

Family PALAEONTINIDAE Handlirsch, 1906

**Genus** ***Hallakkungis*****Nam, Wang & Szwedo, gen. nov.**

**Type species:** *Hallakkungis amisanus* sp. nov.; here designated.

**Diagnosis:** Tegmen with costal margin strongly curved at base. Stem of subcosta posterior (ScP) with several branches intersecting costal area and costal cell, basal portion of ScP shifted from common stem radius + media posterior + cubitus anterior (R + MP + CuA) in distance exceeding the length of basal cell. Stem MP bifurcated into branches MP_1+2_ and MP_3+4_ earlier than stem ScP + R forking. Stem of CuA straight; branch CuA_2_ strongly curved mediad in median ⅓ of its length. Crossvein *r-mp*_*1*_ apicad of crossvein *mp*_*3*+*4*_*-cua*; crossvein *mp*_*3*+*4*_*-cua* connected with stem CuA before CuA forking; crossvein *mp*_*3*+*4*_*-cua* forms part of nodal line; apex of clavus obtuse, due to strong curving of the utmost distal part of cubitus posterior (CuP) claval veins postcubitus (Pcu) and analis prima (A_1_) fused for a short distance as common stalk.

**Remarks:** Based on some venational characters, e.g. anterior margin indented, costa posterior (CP) present and ScP with several branches, this new genus is similar to *Fletcheriana* Evans, 1956, which was reported from the Middle Triassic of Australia. However, the genus *Fletcheriana* Evans, 1956, with *Fletcheriana triassica* Evans, 1956 (New South Wales, Australia) was transferred to Dunstaniidae^16^. In the same paper^16^ the species ‘*Fletcheriana*’ *magna* Riek, 1976, from the Triassic of South Africa^17^ was placed in the family Palaeontinidae. Later, the genus was discussed and some Jurassic species previously ascribed to this genus, were transferred to the other Palaeontinidae genera^14^. The genus *Asiocossus* Becker-Migdisova, 1962 from Kirghizstan^18^ is incompletely preserved (only basal portion of forewing), and the deposit was reevaluated as early Jurassic.

The new genus described above clearly differs from ‘*Fletcheriana*’ *magna* by the very strong curving of costal margin at base, distinct shift of basal part of ScP from common stem R + MP + CuA for a distance exceeding the length of basal cell (this portion is not clear in ‘*Fletcheriana*’ *magna*); stem MP forked anteriad of stem ScP + R forking (similar pattern, but less anteriad in ‘*Fletcheriana*’ *magna*); straight stem CuA (strongly curved in ‘*Fletcheriana*’ *magna*); distinct mediad curving of median portion of CuA_2_ branch (terminal CuA_2_ almost straight in ‘*Fletcheriana*’ *magna*); veinlet *mp*_*3*+*4*_*-cua* composed to nodal flexion line (only part close to CuA_2_ of *mp*_*4*_*-cua* composed to nodal flexion line in ‘*Fletcheriana*’ *magna*; this veinlet meets terminal MP_4_ in ‘*Fletcheriana*’ *magna* not the branch MP_3+4_); *mp*_*3*+*4*_*-cua* fused to stem CuA basad of CuA forking (connected with CuA_1_ slightly apicad of forking in ‘*Fletcheriana*’ *magna*); discal cell about three times as long as wide and narrow (discal cell about twice as wide as long in ‘*Fletcheriana*’ *magna*).

**Etymology:** The generic name is derived from “Hallakkungi” – the Flower Warden God in the Soch’on Flower Garden, from the Korean mythology. Gender: masculine, 3^rd^ declension.

***Hallakkungis amisanus*** **Nam, Wang & Szwedo, sp. nov.**

(Fig. 2a,b).

**Diagnosis:** Forewing elongately triangular, costal margin blade-like, distinct ambient vein and narrow appendix present; corrugations exceeding to narrow appendix and apical portions of apical cells. Stem CP faint, costal area widest at base, with intersecting branchings of ScP more distinct. Stem ScP emitting six braches intersecting costal area and costal cell, these branches dispersed in increasing distance each other. Veinlet *mp*_*3-4*_*-cua* long, slightly sigmoid, connecting branch MP_3+4_ just after its separation from stem MP to stem CuA at ½ of stem CuA length. Discal cell elongately almond-shaped, with acute apical angle, about 3 times as long as broad at widest point.

**Etymology:** The specific epithet is derived from the Amisan Formation, in which the fossil has been found.

**Holotype:** Single right forewing (tegmen), No. KNU-2009018. Deposited in Department of Earth Science Education, Kongju National University, Gongju, Korea.

**Type locality, formation and age:** Boryeong City, South Korea (N36°21′, E126°40′); Amisan Formation, Late Triassic.

**Description:** Right tegmen, 46 mm long, 21 mm wide. Anterior margin strongly curved at base, with distinct nodal incision basad of half of anterior margin length. Anteroapical angle acute, posteroapical angle of 121°; posteroapical margin straight; postclaval margin straight, curved towards apical angle; posteroclaval margin straight. Apex of clavus not reaching ⅓ of total length of forewing. Posteroapical margin corrugated, corrugations on appendix and at basal portions of apical cells. Costal margin (costa anterior; CA) strongly curved at base, blade-like, arcuate to nodal incision, arcuate apicad of nodal incision towards the anteroapical angle. Vein CP obscure, slightly curved and ending at the level of nodal indentation. Stem ScP distinctly separated from common stem R + MP + CuA at base, fused with stem R distinctly apicad of basal cell apex. Costal area and costal cell intersected by six branchings of ScP, with spaces between branchings sequentially increasing; apical portions of these branchings more distinct on costal area. Stem R + MP + CuA thick, stems R, MP and CuA leaving basal cell separately. Stem R strongly curved anteriad at base, forked basad of nodal line incision, slightly posteriad of stem MP forking; branch RA forked basad of nodal line incision, terminal ScP short, branch RA_1_ reaching anterior forewing margin at about half of post-incision portion length, branch RA_2_ longer, reaching anterior margin distinctly basad of anteroapical angle. Stem MP curved at base, forked basad of stem R forking, apicad of stem CuA forking; branch MP_1+2_ shorter than branch MP_3+4_; forking of branch MP_1+2_ merely apicad of nodal incision level, slightly earlier than forking of MP_3+4_; forking MP_3+4_ more apicad than nodal incision; terminals MP_1_, MP_2_, MP_3_ and MP_4_ slightly curved, reaching margin in median portion of posteroapical margin of forewing. Stem CuA leaving basal cell thick, straight, forked at basal ¼ of forewing length, basad of claval apex; branch CuA_1_ curved anteriad, reaching the posteroapical margin before posteroapical angle; branch CuA_2_ straight at basal ⅓, then strongly curved mediad, apical ⅓ thinner, slightly wavy, reaching posterior margin beside the posteroapical angle. Claval vein CuP thicker at base, thinner in apical portion, distinctly curved posteriad at claval apex, forming obtuse claval apex. Claval veins Pcu and A_1_ fused in apical ¼ of clavus. Posteroclaval margin distinct, not strongly separated from postclaval margin. Crossvein *r-mp*_*1*_ short, distinctly apical of nodal line; crossveins *mp*_*3*+*4*_*-cua* long, sigmoid, included to nodal (flexion) line, connecting branch MP_3+4_ slightly after stem MP forking with stem CuA at half of its length after separation from basal cell. Nodal line distinct, from nodal line incision at anterior margin, through terminal ScP, stem R forking, cutting branches MP_1+2_ and MP_3+4_ slightly after the stem MP forking, then, followed with crossvein *mp*_*3*+*4*_*-cua* for a long interval and after separating from *mp*_*3*+*4*_*-cua* crossvein, fused with basal ⅓ of branch CuA_2_ finally, separating from CuA_2_ and reaching CuP at level of claval apex.

## Discussion

*Hallakkungis amisanus* gen. et sp. nov. is the first palaeontinid described from the Korea. Regarding its strongly triangular shape of the forewing, the new taxon resembles more the Early Cretaceous representatives of the family^19^. The more triangular shape of the forewing is an important character of the Palaeontinidae from the Late Jurassic to the Early Cretaceous^20^,^21^,^22^, but the venation pattern is clearly different. In the Jurassic and Cretaceous Palaeontinidae with triangular wings the hind wing is usually diminished due to particular kind of flight, discussed in ref. 23, but the question of a similar tendency in *Hallakungis* gen. nov. hind wings and flight performance remains open. The new genus *Hallakkungis* gen. nov. presents number of unique features, some of them shared also with presumed ancestors of Palaeontinidae, i.e. representatives of the family Dunstaniidae^14^. The strong curve of the costal margin at the base is one of peculiar features of the newly described genus. Such trend in forewing shape is observed in Dunstaniidae (e.g. *Fletcheriana triassica*), also in not related to Palaeontinoidea representatives of superfamily Pereborioidea – families Perboriidae and Ignotalidae^24^,^25^. The very late separation of RA and RP only just before the node, is similar to the situation in the Permian Prosbolidae. Such a late separation is common in Triassic palaeontinoids, and it is probably a plesiomorphic condition, but in *Hallakungis* gen. nov., it is unusually late. Another feature worth of mention is the basal separation of the ScP – such situation is present in majority of Jurassic Palaeontinidae, much less common in Dunstaniidae. The feature resembling the situation common with Dunstaniidae is the connection of crossvein *mp*_*3*+*4*_*-cua* to the stem CuA. In the vast majority of Palaeontinidae it is connected near, at the point or the distad of point of forking of stem CuA, while in *Hallakkungis* gen. nov. this point is distinctly more basal, at half of the common stem CuA length. In the other Palaeontinidae, this crossvein meets the terminal MP_4_, while in *Hallakungis* gen. nov., it is placed more basad, and meeting the branch MP_3+4_ in proximity of stem M forking, which is unusual. The exceptional feature of the newly described genus *Hallakkungis* is that the vast portion of crossvein *mp*_*3*+*4*_*-cua* is involved in forming the nodal line – while nodal line is more proximal then crossvein between in any other palaeontinoids. So, the evolutionary tendency of shifting to more apical position of this connection point is observed among the representatives of Palaeontinidae. It is interesting that the claval veins Pcu and A_1_ are fused in apical ¼ of the clavus length. This is a very ancient feature of early Cicadomorpha, present among the Permian representatives of the suborder, but also in the Triassic Dunstaniidae^16^. It seems that line of claval margin is rather continued on postclaval margin (the specimen is damaged at this area suggesting the incision); the incisions suggested at this point in the Dunstaniidae^17^, seems to be an artifact of preservation. However, the obtuse angle of the clavus is a unique feature of the *Hallakkungis* gen. nov. Another remarkable feature of this fossil is the strong curving in median ⅓ of the branch CuA_2_, such a character is not known among the other Palaeontinidae. It is interesting, that the corrugation at margin of the forewing is found also in some Early Cretaceous Palaeontinidae^21^, but such a feature is present also in taxa not related to the Palaeontinioidea representatives of the Cicadoidea: Cicadidae and Cercopoidea: Cercopidae^26^,^27^.

The newly described above genus and species presents a mixture of plesiomorphic, ancestral features shared with the Triassic Dunstaniidae, apomorphic, derived features found in the Jurassic and Cretaceous palaeontinids, and strongly autapomorphic features. General trends could be observed: the narrowing of the costal lobe, the progressively proximal separation of RA and RP, the development of a long basal crossvein between MP and CuA, so that the basal part of the wing comes to be supported by a three pronged fork of ScP + RA, RP and MP, rather than ScP + RA + RP, MP and CuA. The abovementioned features places this specimen among the unique taxa. *Hallakkungis* gen. nov., is an important link to understanding the evolutionary trends, tendencies and traits of early Palaeontinidae.

This new fossil genus of Palaeontinidae comes from the deposits of the Late Triassic Daedong flora, which is a typical representative of the *Dictyophyllum-Clathropteris* flora of Asia. It contains a number of plant taxa typical of the Triassic or to be relicts of the Permian floras^10^. The great reconstruction of the face of the Earth and of the organic world (the appearance of a great number of new plant forms) started in the Middle Triassic, and has been completed to the end of the Triassic. This distributional pattern of plants remained relatively stable during the rest of Mesophytic. Hence, this could be the source of evolutionary success of phytophagous, phloem-feeding Palaeontinidae.

The new discovery presented above is also the first record of the family Palaeontinidae from the Upper Triassic of Korea. We anticipate discovery of more well-preserved specimens from South Korea that will allow us to carry out more morphological and taxonomic studies, as well as palaeoecological, palaeobiogeograpical and evolutionary analyses of the Palaeontinidae and its relatives.

## Additional Information

**How to cite this article**: Nam, K. S. *et al*. An extraordinary palaeontinid from the Triassic of Korea and its significance. *Sci. Rep.* **7**, 40691; doi: 10.1038/srep40691 (2017).

**Publisher's note:** Springer Nature remains neutral with regard to jurisdictional claims in published maps and institutional affiliations.

## Figures and Tables

**Figure 1:**
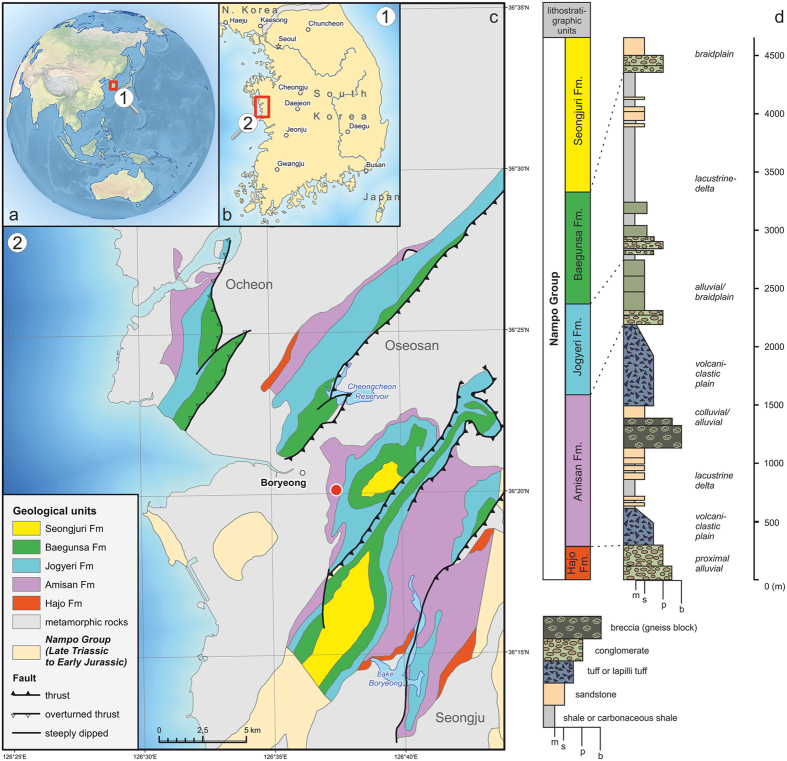
Map showing the placement of fossil insect findings on the geological background of the area. (**a**) location of the fossil site (boxed); (**b**) close-up of the southern Korean Peninsula showing the study area (boxed); (**c**) geological map of study area; (**d**) Strato-sedimentological interpretation of the Nampo Group in the Oseosan subbasin. Figures in inserts a and b were made with data from Natural Earth (http://www.naturalearthdata.com); coastline in c adopted from Open Street Map, with cartography map tiles licensed under CC BY-SA (www.openstreetmap.org/copyright). Figure created using ArcGIS 10.1, CorelDraw X7 and Adobe Photoshop CS software packages.

**Figure 2:**
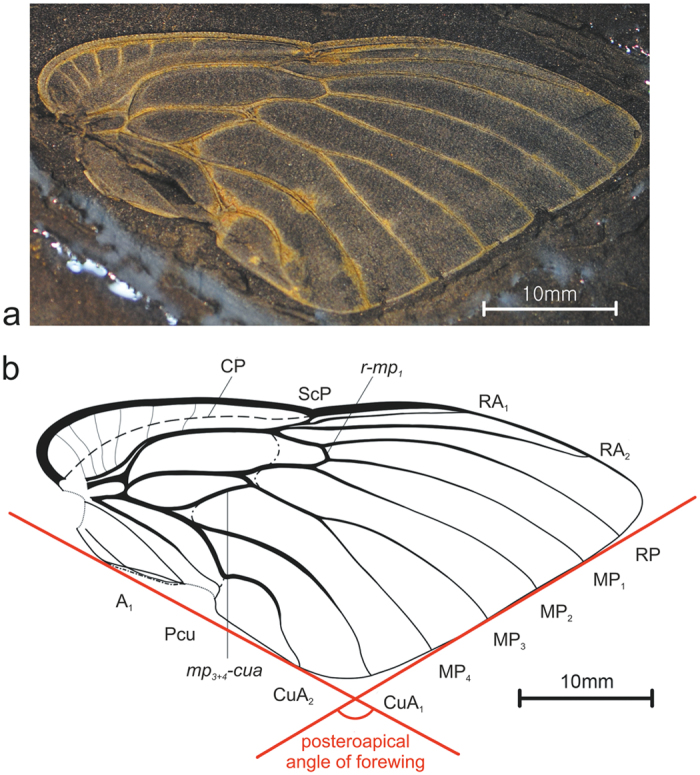
*Hallakkungis amisanus* Nam, Wang et Szwedo, gen. et sp. nov. (**a**) photograph of holotype, No. KNU-2009018, part; (**b**) line drawing of forewing, No. KNU-2009018, drawn on photograph with Adobe Photoshop CS and adjusted with CorelDraw X7 software packages.
